# Demographic variation in space and time: implications for conservation targeting

**DOI:** 10.1098/rsos.211671

**Published:** 2022-03-30

**Authors:** Catriona A. Morrison, Simon J. Butler, Jacquie A. Clark, Juan Arizaga, Oriol Baltà, Jaroslav Cepák, Arantza Leal Nebot, Markus Piha, Kasper Thorup, Thomas Wenninger, Robert A. Robinson, Jennifer A. Gill

**Affiliations:** ^1^ School of Biological Sciences, University of East Anglia, Norwich Research Park, Norwich NR4 7TJ, UK; ^2^ British Trust for Ornithology, The Nunnery, Thetford IP24 2PU, UK; ^3^ Department of Ornithology, Aranzadi Sciences Society, Zorroagagaina 11, E20014 Donostia, Spain; ^4^ Catalan Ornithological Institute, Nat-Museu de Ciències Naturals de Barcelona, Pl. Leonardo da Vinci, 4-5 08019 Barcelona, Spain; ^5^ Bird Ringing Centre, National Museum, Hornoměcholupská 34, CZ-10200 10 Praha, Czech Republic; ^6^ SEO/BirdLife, Ciencia Ciudadana, C/Melquiades Biencinto, 34 - 28053 Madrid, Spain; ^7^ Finnish Museum of Natural History – LUOMUS, P. O. Box 17, FI-00014 University of Helsinki, Finland; ^8^ Center for Macroecology, Evolution and Climate, Natural History Museum of Denmark, University of Copenhagen, Universitetsparken 15, DK-2100 Copenhagen, Denmark; ^9^ Swedish Museum of Natural History, Bird Ringing Centre, Box 50007, S-104 05 Stockholm, Sweden

**Keywords:** conservation actions, demography, productivity, survival rates, population declines

## Abstract

The dynamics of wild populations are governed by demographic rates which vary spatially and/or temporally in response to environmental conditions. Conservation actions for widespread but declining populations could potentially exploit this variation to target locations (or years) in which rates are low, but only if consistent spatial or temporal variation in demographic rates occurs. Using long-term demographic data for wild birds across Europe, we show that productivity tends to vary between sites (consistently across years), while survival rates tend to vary between years (consistently across sites), and that spatial synchrony is more common in survival than productivity. Identifying the conditions associated with low demographic rates could therefore facilitate spatially targeted actions to improve productivity or (less feasibly) forecasting and temporally targeting actions to boost survival. Decomposing spatio-temporal variation in demography can thus be a powerful tool for informing conservation policy and for revealing appropriate scales for actions to influence demographic rates.

## 1. Introduction

The recent Intergovernmental Science-Policy Platform on Biodiversity and Ecosystem Services (IPBES) report delivered a stark warning on the health of global biodiversity—a million species at risk of extinction, with the average abundance of native species in most major land-based habitats having fallen by at least 20% since 1990 [1]. Declines in widespread and common species are increasingly prevalent [1,2] and, so far, the success of intervention methods has been limited [3–5]. Addressing these declines requires a radical, transformative change in the design and delivery of conservation action.

Conservation actions to reverse population declines ultimately aim to influence demography, through the creation of conditions which enhance productivity and/or survival. Most conservation actions are deployed spatially, for example through long-term management of protected areas. However, temporal deployment also occurs, for example, through drought management plans to maintain wetlands in dry years [6], or winter food enhancement to maintain farmland bird populations [7]. The need for better targeting of these management options is increasingly being recognized [8,9], but our ability to influence productivity and survival rates is hampered by our lack of understanding of the spatial and temporal scales over which these rates vary.

**Figure 1.**
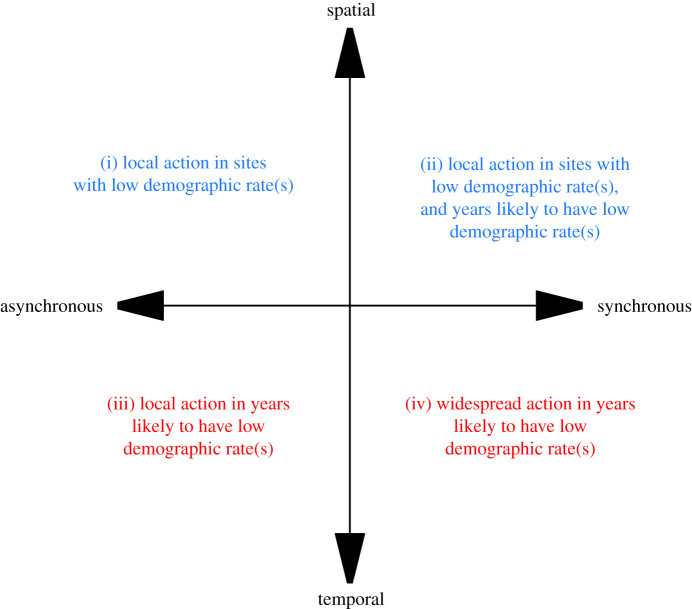
Demographic variation and the appropriate targeting of conservation actions. When demographic rates vary more between sites than years (blue; spatial > annual), conservation actions to improve local conditions at poor sites are likely to be the most effective way of influencing demography (i, ii). When demographic rates vary more between years than sites (red; annual > spatial), annual targeting of conservation action in poor years is likely to be the most effective way of influencing demography (iii, iv). The spatial scale over which actions need to be implemented will vary depending on the strength of synchrony in the demographic rates. Strong synchrony will require local (ii) or widespread (iv) actions to mitigate against poor years, whereas asynchrony (little or no spatial synchrony in annual variation) in demographic rates will require local actions targeted at poor years (iii) and sites (i).

Consistent spatial variation in demographic rates in declining populations would provide a potential platform for the identification of conditions associated with high and low demographic rates. In turn, this could guide the design and targeted delivery of strategies and actions to increase the frequency of sites capable of facilitating high demographic rates (figure 1). However, if demographic rates exhibit more temporal than spatial variation, then conservation actions to boost them would depend on whether periods with low demographic rates could be predicted sufficiently far in advance to be able to deploy counteracting or compensatory measures to boost rates during those periods [10]. The scale of deployment of such actions would be informed by the degree of spatial synchrony in annual demographic variation (e.g. strong synchrony requires widespread actions to address or mitigate low rates, while weak synchrony requires targeted actions in particular locations and years likely to experience low rates).

Quantifying the spatio-temporal structure (STS) of demographic rates, and the extent to which temporal variation in rates is correlated across sites, requires long-term data spanning broad spatial scales of the type generated by citizen-science monitoring schemes [11–15]. Across Europe, constant effort bird ringing schemes (Euro-CES) operate during the breeding season, and the resulting capture-recapture data allow rates of survival and productivity to be quantified [16].

Here we characterize the STS in demographic rates for 26 European breeding bird species, using data from European Constant Effort Scheme (CES) sites. To identify the rates and scales likely to be most suitable as targets for conservation actions, we compare the extent to which productivity and survival rates vary between sites and years, and the extent to which annual variation in these rates is spatially synchronous.

## 2. Methods

### 2.1. Estimating demographic rates from European constant effort site schemes

For 26 passerine bird species (electronic supplementary material, table S1), demographic data were collated from 334 CES sites across eight countries within Europe, all of which use standardized mist-netting during the breeding season to measure the relative annual productivity and apparent survival [16] (electronic supplementary material, table S2). At each site, licensed ringers deploy a series of mist-nets in the same positions, for the same length of time, during morning and/or evening visits, typically between April–May and July–August (the season starts and ends later at higher latitudes). Our analyses included: (i) sites running for 5 or more years, (ii) years in which a site was visited eight or more times in the season, and, for each species, (iii) sites on which 25 or more adults and 25 or more juveniles had been captured in total, between 2004 and 2014. The geographical distribution of these sites within Europe can be seen in the electronic supplementary material, figure S1. Apparent survival estimates can be influenced by individuals dispersing and thus being less likely to be recaptured, but annual variation in dispersal is typically small [17]. For each species, across these sites and years, we ran six models, three each for apparent survival and productivity. The structure of each model is outlined below (equations (2.1), (2.2) and (2.3): electronic supplementary material, table S3). We fitted all models using Bayesian inference implemented in JAGS v. 3.3.0, via the R package rjags [18] (electronic supplementary material), and all subsequent analysis was conducted using R v. 3.4.2 ([19], see https://osf.io/pf2t4/ [20] for the R and JAGS code supporting these analyses).

### 2.2. Quantifying the spatio-temporal structure and synchrony of demographic rates

To characterize the structure and synchrony of spatio-temporal variation in demographic rate (either apparent survival or productivity, for which one value is available for each site in each year), we constructed two Bayesian models from the CES data and used their outputs to calculate two separate metrics: (i) STS: the proportion of the total demographic variation (spatial and annual) that is spatial (among site), and (ii) interclass correlation coefficient (ICC): the ratio of the temporal variability that is common across all populations (synchronous) to the total spatio-temporal variability [21,22]. STS captures the spatial proportion of the spatio-temporal demographic variation, while the ICC captures the degree of spatial synchrony in annual variation, as follows.

### 2.3. Spatial-temporal structure

For each species, we decomposed the variation in each demographic rate into its global spatial and global temporal components using random effect models (equation (2.1)):

2.1 $$g(\vartheta_{k,t})=\mu +\varepsilon_{t}+{ŋ}_{k},$$

where *g* is the logit link function, *µ* is the overall mean of ϑ (demographic rate) on the scale of the link function, *ɛ_t_* represents annual variation and ŋ*_k_* represents the site-level variation. The variation is modelled with normal distributions where *ɛ_t_* ∼ *N*(0,*T*^2^) and ŋ*_k_* ∼ *N*(0,*K*^2^), where *T*^2^ is the annual variation and *K*^2^ is the site-level variation. We then calculated the *STS* estimate for each demographic rate as:

2.2 $$\mathrm{STS}=\frac{K^{2}}{T^{2}+K^{2}}.$$

A value of STS < 0.5 indicates that annual variation is greater than site-level variation and a value greater than 0.5 indicates that site-level variation is greater than annual variation.

### 2.4. Interclass correlation coefficient

For each species, we decomposed the temporal variation in each demographic rate into a global component which is common to all sites (a synchronous part) and a local component that varies among sites (an asynchronous part) using random effects models [22]:

2.3 $$g(\vartheta_{k,t})=\mu_{k}+\varepsilon_{t}+{ŋ}_{k,t},$$

where *g* is the logit link function, *µ_k_* is the mean of ϑ (demographic rate) in site *k* on the scale of the link function, *ɛ_t_* represent annual fluctuations that are shared across all populations, and ŋ*_k,t_* expresses differences in annual fluctuations among populations. To decompose the total variation into its global and site-specific components, temporal deviations are modelled with normal distributions and thus *ɛ_t_* ∼ *N*(0,*T*^2^) and ŋ*_k,t_* ∼ *N*(0,*P*^2^), where *T*^2^ is the global variability and *P*^2^ is the site-specific (i.e. local) variability not explained by global variability [21,22]. We then calculated the ICC estimate for each demographic rate as

2.4 $$\mathrm{ICC}=\frac{T^{2}}{T^{2}+P^{2}}.$$

An ICC close to 1 indicates strong spatial synchrony (annual variation is consistent across sites) while an ICC close to 0 indicates spatial asynchrony (annual variation differs across sites).

To assess the mean difference between the STS for apparent survival and productivity, we sub-sampled the posterior distributions generated by the Bayesian modelling procedures to calculate 700 differences (productivity STS – survival STS) for each species (700 was the minimum number of iterations for any species). For each iteration, we then took the mean of these differences across all 26 species as the overall mean difference. Significant differences (between productivity STS and survival STS) were identified as those in which the 97.5th and 2.5th quantiles of the distribution of mean differences did not overlap zero. This method was repeated to assess differences in synchrony (ICC) between each rate.

## 3. Results

### 3.1. Spatio-temporal structure and synchrony of demographic rates

**Figure 2.**
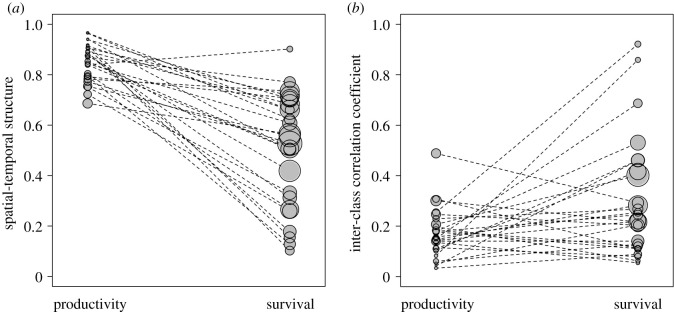
Differences between productivity (juveniles per adult) and survival (annual adult rates) in their (*a*) STS(0 = wholly annual; 1 = wholly spatial) and (*b*) ICC (0 = asynchronous; 1 = synchronous) across 26 bird species breeding in Europe. Dashed lines join each species' estimates. Point size varies relative to the standard deviation of each estimate (smallest = 0.03, largest = 0.36).

Across the 26 species, productivity varies more between sites than between years (STS > 0.5), and these STS values were significantly higher than for apparent survival rates (figure 2*a*, mean difference = 0.35, 0.025th and 0.975th percentiles = 0.25 and 0.44, respectively). Thus, sites tend to have consistently high or low productivity while apparent survival tends to show more annual variation across sites; for about half of the species, apparent survival varies more between years than sites (STS < 0.5). There was no effect of the number of years or sites over which each species was recorded on the STS in productivity or apparent survival rates (electronic supplementary material, figure S2).

Spatial synchrony is significantly lower in productivity than apparent survival rates across these species (figure 2*b*, mean difference = −0.13, 0.025th and 0.975th percentiles = −0.06 and −0.21). Thus, the relatively small amount of annual variation in productivity varies among sites (good years at one site are not necessarily good years elsewhere) while the larger amount of annual variation in apparent survival rates is more consistent among sites (good years at one site are more likely to be good years elsewhere). However, the degree of synchrony (ICC) is still less than 0.5 in 22 of the 26 species, indicating variation in where and when good years for survival occur for many species (figure 2*b*).

**Figure 3.**
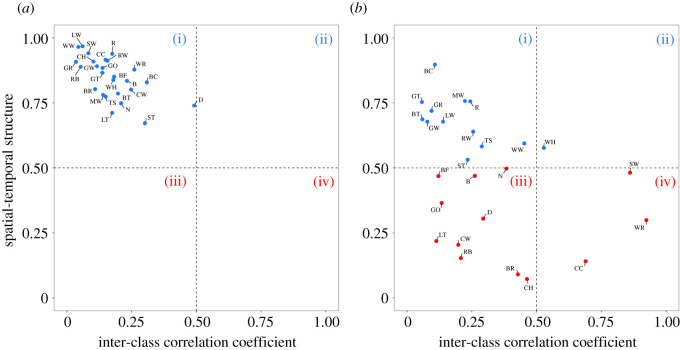
The relationship between STS and the ICC for (*a*) productivity (*b*) the ICC for survival. Letters indicate British Trust for Ornithology species codes (electronic supplementary material, table S1). Roman numerals correspond to the quadrants of figure 1.

Productivity in all species exhibits primarily spatial variation (high STS) that is asynchronous (low ICC, figure 3*a*; productivity varies more between sites than between years and variation between years is inconsistent among sites) and is therefore in the quadrant in which targeted local action in sites with low productivity is likely to be most effective (figure 1(i)). However, the structure of spatial and temporal variation in apparent survival rates is much more variable between species: 12 of the 26 species show high STSs and low ICCs (figure 3*b*, quadrant (i)), while 10 have low STSs and low ICCs (figure 3*b*, quadrant (iii); survival varies more between years than between sites and variation between years is inconsistent among sites), suggesting that targeted local action in either sites (i) or sites and years (iii) with low survival rates would be most effective (figure 1). Three species (*Phylloscopus collybita* (chiffchaff), *Troglodytes troglodytes* (wren) and *Acrocephalus schoenobaenus* (sedge warbler), have low STSs and high ICCs in survival rates (figure 3*b*, quadrant (iv); more inter-annual than inter-site variation, and inter-annual variation is spatially synchronous), suggesting that widespread action to boost survival rates in poor years may be most effective. Only one species, *Sylvia communis* (whitethroat), has slightly more spatial than temporal variation together with some degree of synchrony in survival rates (ICC = 0.52, figure 3*b*, quadrant (ii); more inter-site than inter-annual variation, and inter-annual variation is spatially synchronous).

## 4. Discussion

Deploying conservation actions in the correct sites, years and across suitable spatial scales, to reduce the frequency of poor conditions in space and time, is likely to enhance both the effectiveness and cost-effectiveness of conservation strategies. Consistent spatial variation in demographic rates will greatly facilitate spatial targeting of actions, while targeting of annual variation will require identification of the conditions that are likely to lead to periods with low demographic rates, sufficiently far in advance to allow deployment of actions.

In all 26 species studied, productivity varied more between sites than years and had weak spatial synchrony, with no clear clustering of species with differing migratory status or current population trends. CES sites are broadly distributed across Europe, across species ranges (electronic supplementary material, figure S1). It is therefore unlikely that the spatial distribution of the sites included in our analysis will influence the main conclusions of this study. Our findings strongly suggest that local-scale environmental conditions are key drivers of variation in productivity. Previous studies have linked regional variation in population trends across Europe (1970–1990) to large-scale changes in farming practices [23,24]. However, high levels of local-scale variation in population trends have also been found [25], which could reflect local-scale variation in environmental processes such as habitat degradation or fragmentation [26]. Such processes can, for example, lower food availability and increase vulnerability to nest predation [27] and therefore have the potential to greatly influence local productivity. Improving productivity as a means to address population declines is therefore likely to require actions targeted at influencing local environmental conditions in the most degraded sites, and these findings suggest that such actions could potentially benefit whole communities breeding at these sites.

By contrast, apparent survival rates tended to vary more between years than sites. Given that most individuals are only present on breeding sites for a few weeks in each year, and that the rest of the year could be spent anywhere from the surrounding landscape to locations across Europe or Africa, strong spatial consistency in survival rates at breeding sites would only be expected if breeding season conditions were the primary driver of mortality. Individuals breeding at the same sites could experience similar environmental conditions (e.g. severe weather conditions) during the non-breeding season but, particularly among migratory species, this should result in large-scale synchrony in survival variation, as those conditions would inevitably influence individuals from many different breeding locations [28]. Strong evidence of temporal synchrony in survival rates was apparent in three species only (figure 3*b*(iv)). Wren and chiffchaff are both small, insectivorous species and therefore sensitive to changes in prey availability during harsh winters [29,30], while the survival rates of sedge warblers have historically been linked to periods of drought across the Sahel region [31]. These findings indicate that survival rates are influenced by conditions that vary annually and sometimes over large areas and therefore actions targeted at influencing local environmental conditions as a means to increase survival rates are likely to be ineffective.

Our analyses of land-birds breeding across Europe demonstrate that context-dependent-targeted action to boost demographic rates is required, and that spatial targeting to boost productivity (in sites where it is consistently low) is likely to be more appropriate (and feasible) than temporal forecasting and targeting to boost survival rates (in years in which it is likely to be low). Whether such targeting of actions would be sufficient to reverse population declines will depend on our capacity to identify the environmental conditions that contribute to the spatial and temporal variation in demography and deploy appropriate actions at sufficient scales. Citizen-science programmes, for birds and other taxa, can play a key role in the design and delivery of targeted actions to boost demographic rates and thus in addressing the growing challenge of conserving widespread but declining species.

## Data Availability

The R and JAGs code and data supporting this article are available from: https://osf.io/pf2t4/ [20].
